# An oscillating reaction network with an exact closed form solution in the time domain

**DOI:** 10.1186/s12859-023-05600-w

**Published:** 2023-12-09

**Authors:** Joseph Hellerstein

**Affiliations:** eScience Institute, University of Washington, Seattle, USA

**Keywords:** Systems biology

## Abstract

**Background:**

Oscillatory behavior is critical to many life sustaining processes such as cell cycles, circadian rhythms, and notch signaling. Important biological functions depend on the characteristics of these oscillations (hereafter, oscillation characteristics or OCs): frequency (e.g., event timings), amplitude (e.g., signal strength), and phase (e.g., event sequencing). Numerous oscillating reaction networks have been documented or proposed. Some investigators claim that oscillations in reaction networks require nonlinear dynamics in that at least one rate law is a nonlinear function of species concentrations. No one has shown that oscillations can be produced for a reaction network with linear dynamics. Further, no one has obtained closed form solutions for the frequency, amplitude and phase of any oscillating reaction network. Finally, no one has published an algorithm for constructing oscillating reaction networks with desired OCs.

**Results:**

This is a theoretical study that analyzes reaction networks in terms of their representation as systems of ordinary differential equations. Our contributions are: (a) construction of an oscillating, two species reaction network [two species harmonic oscillator (2SHO)] that has no nonlinearity; (b) obtaining closed form formulas that calculate frequency, amplitude, and phase in terms of the parameters of the 2SHO reaction network, something that has not been done for any published oscillating reaction network; and (c) development of an algorithm that parameterizes the 2SHO to achieve desired oscillation, a capability that has not been produced for any published oscillating reaction network.

**Conclusions:**

Our 2SHO demonstrates the feasibility of creating an oscillating reaction network whose dynamics are described by a system of *linear* differential equations. Because it is a linear system, we can derive closed form expressions for the frequency, amplitude, and phase of oscillations, something that has not been done for other published reaction networks. With these formulas, we can design 2SHO reaction networks to have desired oscillation characteristics. Finally, our sensitivity analysis suggests an approach to constructing a 2SHO for a biochemical system.

**Supplementary Information:**

The online version contains supplementary material available at 10.1186/s12859-023-05600-w.

## Background

Oscillatory behavior is critical to many life sustaining processes. Examples include: cell cycles [1], circadian rhythms [2], notch signaling in the development of the nervous system [3], tissue development [4], gene transcription [5], and efficient signaling [6]. Biological oscillators are also important elements in building applications in synthetic biology [7–9].

The characteristics of biological oscillations often have critical biological functions. Frequency is used to control the times at which events are initiated, such as circadian cycles and chromatin modifications [10]. Amplitude controls the strength of signaling [11]. Phase plays a role in the sequencing of processes within the cell cycle [12]. Since biological oscillators typically cause changes in the concentration of chemical species, these oscillators must have a DC offset so that values are non-negative. Collectively, we refer to frequency, amplitude, phase, and DC offset as **oscillation characteristics (OCs)**.

One way to understand the relationship between an oscillating reaction network and its OCs is to construct a closed-form, time-domain solution of the network’s behavior in terms of parameters such as kinetic constants and initial concentrations of chemical species. From these mathematical expressions, we obtain insights such as: (a) if one or more reactions are unnecessary to achieve oscillations; (b) relationships between the kinetic constants of reactions; and (c) how to assign values to network parameters so as to achieve desired OCs.

Many researchers have investigated structural aspects of oscillating reaction networks. These structures include: positive feedback, negative feedback, balancing reaction rates, and ultrasensitivity [13–17]. Others have built biological oscillators [7, 8, 12, 18–21]. But neither kind of investigation addresses our interest in a closed-form, time-domain solution that relates parameters of a reaction network to its OCs.

More relevant to our work are quantitative models of biological oscillators. For the most part, existing models are systems of nonlinear ordinary differential equations [22–27]. Typically, these nonlinearities arise because one or more rate law is a nonlinear function of species concentrations (e.g., Michaelis–Menten kinetics). The complexity of these models prohibits the construction of a closed-form, time-domain solution.

We are aware of two approaches that circumvent the limitations of nonlinear ODEs. The first uses an empirical approach, system identification, to construct a linear model that approximates the nonlinear system (e.g., [28]). Models constructed in this way provide accurate predictions near the operating point at which system identification is done. The second approach constructs a linear approximation to a nonlinear ODE (e.g., [29]). Typical approximations make assumptions about relative reaction rates and/or magnitudes of species concentrations. In both cases, the construction of a linear models greatly reduces the complexity of the mathematical expressions, and this in turn makes it possible to obtain a closed-form, time-domain solution. However, the approximations limit the extent to which the resulting mathematical expressions provide useful interpretations of how the parameters of the reaction network affect OCs.

The present work is a theoretical study in that we do not build a biological oscillator. Instead, we consider reaction networks as modelled by systems of ordinary differential equations. Gonze and Ruoff [30] claim that oscillations in reaction networks require that “the kinetic rate laws of the reaction mechanism must be sufficiently ‘nonlinear’ to destabilize the steady state.” This claim is echoed by [31] as well. We note that the context for these claims may be for biological implementations of oscillators. Our work is a theoretical exploration of the construction of an oscillator for a reaction network whose dynamics can be described by a system of linear ordinary differential equations.

It turns out that such a construction is possible. Indeed, we are able to construct a harmonic oscillator for a two species reaction network. We refer to this network as a *two species harmonic oscillator (2SHO)*. Because the 2SHO has linear system dynamics, its behavior depends on initial conditions (e.g., [32]).

At first glance, this may seem to be a modest undertaking since it is well known that harmonic oscillators can be constructed from a system of linear differential equations. Specifically, harmonic oscillations result when the Jacobian matrix of the linear system has pure imaginary eigenvalues (e.g, [33]). So, why not just populate the Jacobian such that the reaction network has imaginary eigenvalues?

Such an approach is difficult to apply to *reaction networks* because of constraints on the system of linear differential equations. Foremost, reaction networks must produce concentrations of chemical species that are non-negative (since negative concentrations are impossible). This turns out to be a non-trivial constraint to satisfy.

A second challenge is that we cannot instantiate a reaction network from an arbitrary Jacobian matrix since the nature of reaction networks imposes constraints on the Jacobian matrix. In particular, a single reaction may affect multiple entries in the matrix. For example, consider a two species reaction network that has a $2 \times 2$ Jacobian, ${\textbf {A}} = \{ a_{ij} \}$. Suppose there is a reaction $S_1 \rightarrow S_2$ with the mass action rate law $kS_1$. This one reaction affects both $a_{11}$ and $a_{12}$; it subtracts $k S_1$ from $a_{11}$, and it adds the same amount to $a_{21}$.

A final challenge is that the choice of rate laws must be biologically plausible. That is, we cannot simply invent a rate law that results in a Jacobian with pure imaginary eigenvalues. In our work, a rate law is biologically plausible if it is used in a published model such as BioModels [34].

The contributions of this paper are:

1. construction of a harmonic oscillator from a two species reaction network (two species harmonic oscillator (2SHO)), a result that demonstrates that an oscillator can be implemented in a reaction network with no nonlinearity;
2. obtaining closed form formulas that calculate the frequency, amplitude, and phase of the 2SHO in terms of its parameters, something that has not been done for any published oscillating reaction network; and
3. development of the parameterizeOscillator algorithm that parameterizes the 2SHO to achieve desired oscillation, a capability that has not been produced for any oscillating reaction network.

We use these results to analyze the sensitivity of 2SHO to variations in values of the kinetic constants; typical varaiabilities are between 1 and 20% (e.g., [35, 36]). Our analysis suggests that with 4 to 6 attempts, there is a 90% probability of constructing a 2SHO for a suitable biochemical system.

## Methods

Our method is to propose a two species reaction network whose kinetics can be described by a system of linear differential equations. We solve this system (an initial value problem) to obtain a harmonic oscillator with closed form formulas that relate OCs to parameters of the reaction network. Finally, we develop an algorithm for parameterizing the two species harmonic oscillator to achieve desired OCs.

### Two species reaction network with linear dynamics

This section develops a biologically feasible reaction network whose kinetics can be described by a system of linear ODEs. Our approach constructs a harmonic oscillator, a model that has been used elsewhere to explain binding and activation between insulin and insulin growth factor receptors [37]. Indeed, harmonic oscillator behaviors are apparent in parameterizations of the Lotka-Volterra and Susceptible Infectious Recovered (SIR) models [38], both of which are widely used in biology.

**Fig. 1:**
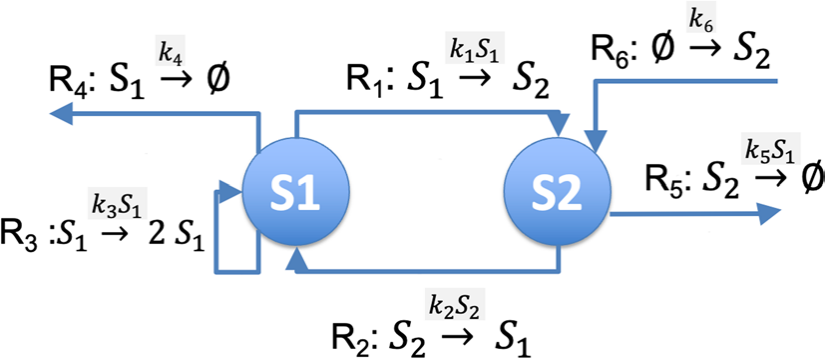
Two species harmonic oscillator (2SHO). Reaction network that creates oscillations in the chemical species $S_1, S_2$. The reaction network is designed so that its time domain solution is a system of linear differential equations. The text describes constraints on the kinetic constants ($k_i$) and initial conditions of the chemical species to create an oscillator such that species concentrations are non-negative, a requirement for biological feasibility

Figure 1 displays our two species harmonic oscillator (2SHO). The construction of 2SHO is driven by the fundamentals of harmonic oscillators in linear systems, as we now describe. The matrix representation of our linear system is:

1 $$\begin{aligned} \dot{{\textbf {x}}}(t) = {\textbf {A}} {\textbf {x}}(t) + {\textbf {u}}, \end{aligned}$$

where ${\textbf {x}} (t) = \{x_n (t)\}$ is an *N* dimension vector of time varying of species concentrations; $\dot{{\textbf {x}}} (t)$ is the time derivative of ${\textbf {x}}(t)$; ${\textbf {A}} = \{a_{ij} \}$ is an $N \times N$ Jacobian matrix of constants; and ${\textbf {u}}$ is an *N* dimensional vector of constants that are forced inputs. We want to construct a reaction network that has a sustained oscillation. Since this is a linear system, the oscillations will be sinusoids (e.g., [32]).

From the foregoing, we have the following constraints:

- **C1**: Rate laws in the reaction network are a linear function of the concentrations of $x_n$(t).
- **C2**: $x_n(t) \ge 0$ so that the reaction network is biologically feasible.

We simplify the problem by having $N=2$ since this is sufficient to obtain oscillations. This means that the eigenvalues of ${\textbf {A}}$ must be pure imaginary numbers (e.g., [39]). Let $\tau = a_{11} + a_{22}$ be the trace of ${\textbf {A}}$, and $\Delta = a_{11} a_{22} - a_{12} a_{21}$ be the determinant of ${\textbf {A}}$. The eigenvalues are complex conjugates $\lambda _1, \lambda _2$ such that

$$\begin{aligned} \lambda _n = \frac{\tau }{2} \pm \frac{\sqrt{\tau ^2 - 4 \Delta }}{2} \end{aligned}$$

Clearly, we obtain pure imaginary eigenvalues only if $\tau = 0$ and $\Delta > 0$. Thus, we add the constraints

- **C3**: $\tau = 0$, where $\tau$ is the trace of ${\textbf {A}}$.
- **C4**: $\Delta > 0$, where $\Delta$ is the determinant of ${\textbf {A}}$.

With these constraints, $\lambda _n = \pm \theta i,$ where $\theta = \sqrt{\Delta }.$

We use $k_i \ge 0$ to specify kinetics constants, where *i* indexes the reaction in the network. Let $S_n$ be a chemical species whose time varying concentration is the state variable $x_n(t)$ for $n=\{1,2\}.$ We start by having two-way interactions between the species. Rate laws are specified above the reaction arrow.

- $$R_1: S_1 \xrightarrow {k_1 S_1} S_2$$
- $$R_2: S_2 \xrightarrow {k_2 S_2} S_1$$

The foregoing reactions have mass action kinetics, which is widely used in models of chemical systems. With just these reactions, ${\textbf {A}}$ is

$$\begin{aligned} \left( \begin{matrix} - k_1 &{}\quad k_2 \\ k_1 &{}\quad -k_2 \end{matrix} \right) \end{aligned}$$

Clearly, C3 does not hold unless we eliminate $R_1, R_2$ by having $k_1 = 0 = k_2$ so that $\tau = 0.$ Instead, we make $a_{11}$ positive by adding an autocatalysis reaction, a kind of reaction that arises in many biological oscillators [40].

- $$R_3: S_1 \xrightarrow {k_3 S_1} 2 S_1$$

and so ${\textbf {A}}$ becomes

$$\begin{aligned} \left( \begin{matrix} k_3 - k_1 &{}\quad k_2 \\ k_1 &{}\quad -k_2 \end{matrix} \right) \end{aligned}$$

which is positive with appropriate choices of the $k_i$.

Since $R_3$ synthesizes $S_1$, we need a reaction that degrades $S_1$ in order for the system to be stable. If we use mass action kinetics for this reaction, $kS_1$, it changes $a_{11}$ to $k_1-k_2-k$, which makes it more difficult to satisfy C3. An alternative is a fixed degradation rate of $k_4 > 0$, where $k_4 = u_1$ is the first element of the vector ${\textbf {u}}$ in Eq. (1).

- $$R_4$$: $$S_1\xrightarrow {k_4}\emptyset$$

Examples of fixed rate degradation reactions in BioModels are: reaction reaction_0 in BIOMD0000000112, reaction ATP_Jerp in BIOMD0000000059, and reaction inhibition_parameter2 in BIOMD0000000224.

We must still address constraint C4, that the determinant is positive. This requires that $a_{12} a_{21} < 0,$ which mean that one of $a_{12}, a_{21}$ is positive and the other is negative. We make $a_{21} < 0$ by degrading $S_2$ at a rate controlled by $S_1$. That is,

- $$R_5$$: $$S_2 \xrightarrow {k_5 S_1}\emptyset$$

Having the degradation of one chemical species controlled by another chemical species is common in BioModels. Some examples are: the fast reaction in model BIOMD0000000108, reaction r10 in BIOMD0000000145, and reaction RuBisCO_5_EOP in BIOMD0000000392. With the addition of reaction $R_5$, ${\textbf {A}}$ becomes

$$\begin{aligned} \left( \begin{matrix} k_3 - k_1 &{}\quad k_2 \\ k_1 - k_5 &{}\quad -k_2 \end{matrix} \right) . \end{aligned}$$

The final reaction in our network compensates for degrading $S_2$ by synthesizing this species at a fixed rate. That is,

- $$R_6$$: $$\emptyset \xrightarrow {k_6} S_2$$

(We do not explicitly cite examples of similar synthesis reactions since they are widely used in BioModels.) From this, we observe that

2 $$\begin{aligned} {\textbf {u}} = \left( \begin{matrix} -k_4 \\ k_6 \end{matrix} \right) . \end{aligned}$$

C3 and C4 constrain the values of the kinetic constants. From C3, we know that

3 $$\begin{aligned} k_3 = k_1 + k_2 \end{aligned}$$

From C4, we know that $(k_3 - k_1)(-k_2) - k_2(k_1 - k_5) > 0,$ or $k_3 < k_5.$ We define $k_d >0$ such that

4 $$\begin{aligned} k_5 = k_3 + k_d \end{aligned}$$

And so, $k_5 = k_1 + k_2 + k_d$. This gives us

5 $$\begin{aligned} {\textbf {A}} = \left( \begin{matrix} k_2 &{} k_2 \\ -k_2 -k_d &{} -k_2 \end{matrix} \right) . \end{aligned}$$

And from this we calculate the determinant of **A**:

6 $$\begin{aligned} \Delta = k_2 k_d . \end{aligned}$$

And hence, the frequency $\theta$ is

7 $$\begin{aligned} \theta = \sqrt{k_2 k_d}. \end{aligned}$$

Further, $k_2, k_d > 0$ implies that

8 $$\begin{aligned} \Delta > 0. \end{aligned}$$

Since $k_3, k_5$ are calculated from other parameters, we refer to them as *dependent parameters*. $k_1, k_2, k_4, k_6, k_d, x_1 (0), x_2 (0)$ are the *independent parameters*.

A brief technical note on Eq. (7). There are actually two solutions for $\theta$, $\pm \sqrt{k_2 k_d}$. These solutions result in oscillations at the same frequency but with different phases. Our approach is to treat phase as a separate oscillation characteristic, and so we just use $\theta$ for the frequency.

We can now satisfy 3 of our 4 constraints. C1 is satisfied since all rate laws are linear in $x_n (t)$, the time varying concentrations of $S_n$. C3 is satisfied since $\tau = k_2 + (- k_2) = 0.$ And, C4 is satisfied by Eq. (8). To address C2, that $x_n(t) \ge 0$, we must find the time domain solution of the reaction network.

### Time domain solution for two species harmonic oscillator

Solving Eq. (1) is an initial value problem, where the initial values of $S_1$, $S_2$ are $x_1(0), x_2(0)$. We proceed as follows: (a) solve the homogeneous equation ${\dot{{\textbf {x}}}}^H = {\textbf {A}} {\textbf {x}}^H(t)$; (b) find a particular solution such that $\dot{{\textbf {x}}}^P (t) = {\textbf {A}} {\textbf {x}}^P(t) + {\textbf {u}}$; and (c) properly structure the complete solution $${\textbf {x}}(t) = \begin{pmatrix} x_1 (t) \\ x_2(t) \end{pmatrix} = {\textbf {x}}^H (t) + {\textbf {x}}^P (t)$$ so that we isolate terms for amplitude, frequency, phase, and DC offset. The derivation is a bit long, and so full details are reported in the Additional file 1.

The solutions for $x_n(t)$ have the form

9 $$\begin{aligned} x_n(t) = \alpha _n cos(t \theta + \phi _n) + \omega _n, \end{aligned}$$

where $\alpha _n$ is the amplitude of oscillation for $x_n(t)$, $\theta$ is the frequency in radians, $\phi _n$ is the phase in radians, and $\omega _n$ is the DC offset. $\alpha _n, \theta , \phi _n, \omega _n$ are functions of the $k_i$ and $x_n (0)$. Table 1 displays the formulas for the OCs. Because of technical details related to the inverse tangent function, $\phi _n$ depends on the term $\pi _n$. (See the Additional file 1 for more on these technical details.) These terms are:

$$\begin{aligned} cond_1= & {} \frac{k_{2}^{2} x_1 (0)}{k_{2} \theta + k_{d} \theta } + \frac{k_{2}^{2} x_2 (0)}{k_{2} \theta + k_{d} \theta } + \frac{k_{2} k_{4} \theta }{k_{2} \theta ^{2} + k_{d} \theta ^{2}} - \frac{2 k_{2} k_{4}}{k_{2} \theta + k_{d} \theta } - \frac{k_{2} k_{6} \theta }{k_{2} \theta ^{2} + k_{d} \theta ^{2}} \\{} & {} + \frac{k_{2} k_{6}}{k_{2} \theta + k_{d} \theta } + \frac{k_{2} k_{d} x_1 (0)}{k_{2} \theta + k_{d} \theta } + \frac{k_{4} k_{d} \theta }{k_{2} \theta ^{2} + k_{d} \theta ^{2}} - \frac{2 k_{4} k_{d}}{k_{2} \theta + k_{d} \theta } + \frac{\theta x_2 (0)}{k_{2} + k_{d}}\\ \pi _1= & {} \pi \text { if } cond_1 < 0 \\= & {} 0 \text{ otherwise } \end{aligned}$$

Similarly,

$$\begin{aligned} cond_2= & {} \frac{k_{2} x_1 (0)}{\theta } + \frac{k_{2} x_2 (0)}{\theta } - \frac{k_{6}}{\theta } + \frac{k_{d} x_1 (0)}{\theta }\\ \pi _2= & {} \pi \text { if } cond_2 > 0 \\= & {} 0 \text{ otherwise } \end{aligned}$$

With the symbolic solution in hand, we return to constraint $C_2$, $x_n (t) \ge 0.$ This is equivalent to $\omega _n \ge \alpha _n.$ Unfortunately, the complexity of the OC formulas makes it difficult to solve these formulas to find constraints on parameters that ensure $C_2$. Indeed, it is frustratingly difficult to find special cases in which C2 is satisfied. For example, consider the situation in which $k_2$ is large. This results in the following:

10 $$\begin{aligned} lim_{k_2 \rightarrow \infty } (\omega _1 - \alpha _1) = sign \left( - k_{4} + k_{6} - T \right) \infty \end{aligned}$$

11 $$\begin{aligned} lim_{k_2 \rightarrow \infty } (\omega _2 - \alpha _2) = sign \left( k_{4} - k_{6} - T \right) \infty \end{aligned}$$

where $T = \sqrt{k_{4}^{2} - 2 k_{4} k_{6} + k_{6}^{2} + \theta ^{2} (x_1 (0))^{2} + 2 \theta ^{2} x_1 (0) x_2 (0) + \theta ^{2} (x_2 (0))^{2}}.$ We can satisfy Eq. (10) (i.e., $x_1 (t) \ge 0$) by making $k_6$ large and $k_4$ small. However, to satisfy Eq. (11) (i.e., $x_2(t) \ge 0$), we must do the opposite: make $k_6$ small and make $k_4$ large.

### Parameterizing the two species harmonic oscillator

Since it is difficult to construct a symbolic expression for the constraint $x_n (t) \ge 0$, we proceed numerically by developing an algorithm that finds values of the independent parameters that achieve desired OCs for the 2SHO. Desired OCs are indicated by the superscript $\star$: $\theta ^{\star }, \alpha ^{\star }, \phi ^{\star }, \omega ^{\star }$.

We proceed by formulating an optimization problem. The objective is to find values of independent parameters that result in the best match with $x^{\star }(t) = \alpha ^{\star } sin(\theta ^{\star } t + \phi ^{\star }) + \omega ^{\star }$. This is done by searching the space of possible values of independent parameters. We denote elements of the search space by ${\mathcal {P}}$, where ${\mathcal {P}}$ is a vector that specifies the value of each independent parameter. We want to find $\hat{{\mathcal {P}}}$ in this space such that $x_n(t; \hat{{\mathcal {P}}})$ is close to $x^{\star }(t)$ for one of $n \in \{1, 2\}.$

Given candidate parameters ${\mathcal {P}}$, we can apply the formulas in Table 1 to calculate $x_n(t; {\mathcal {P}}).$ The quality of this candidate is assessed using the loss function

12 $$\begin{aligned} L_n ({\mathcal {P}}) = \sum _t \left( x^{\star }_n(t) -x_n(t; {\mathcal {P}}) \right) ^2 \end{aligned}$$

This gives us the solutions

$$\begin{aligned} \hat{{\mathcal {P}}}_n= & {} argmin_{{\mathcal {P}}} L_n ({\mathcal {P}}) \\{} & {} \text { such that } x_1 (t; \hat{{\mathcal {P}}_n}), x_2(t; \hat{{\mathcal {P}}_n}) \ge 0 \end{aligned}$$

To simplify our notation in the sequel, ${\hat{x}}_n (t) = x_n(t; \hat{{\mathcal {P}}_n}).$

Our algorithm constructs a single sinusoid. Does it matter whether we use $S_1$ or $S_2$? We refer to the chosen species as the *chosen oscillating species*. It turns out that depending on the desired OCs, sometimes it is better to choose $S_1$; other times $S_2$ works better. The algorithm selects the chosen oscillating species based on which loss function is smaller, $L_1 (\hat{{\mathcal {P}}}_1)$ or $L_2 (\hat{{\mathcal {P}}}_2)$. We denote the chosen oscillating species by ${\hat{x}} (t).$

We observe that the optimization can be simplified in a couple of ways. First, the only constraint on $k_1$ is $k_1 \ge 0.$ So, we set $k_1 = 1$. Second, we can calculate $k_d$ from the desired frequency $\theta ^{\star }$ using Eq. (7), and so $k_d = \frac{(\theta ^{\star })^2}{k_2}$. The combination of these simplifications reduces the dimension of the search space from 7 to 5.

**Fig. 2:**
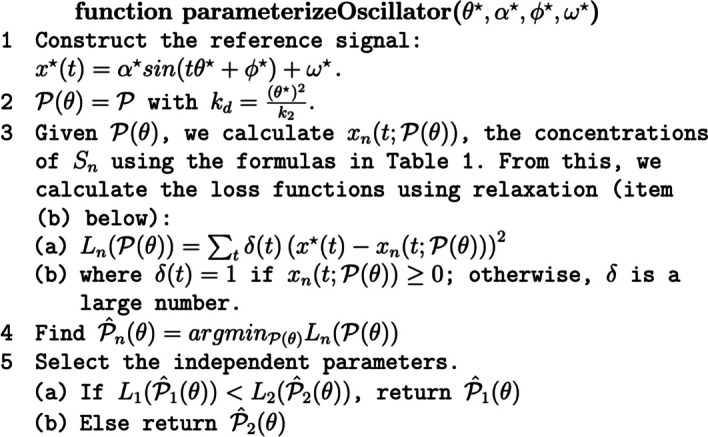
Algorithm for finding values of parameters for the two species harmonic oscillator that achieve desired oscillation characteristics. The function parameterizeOscillator takes as inputs the desired oscillator characteristics and returns the independent parameters ${\mathcal {P}} (\theta )$ for the reaction network: $k_2, k_4, k_6, x_1(0), x_2(0)$. In essence, parameterizeOscillator inverts $x_n (t)$ by finding the ${\mathcal {P}}$ that minimizes the squared error difference between the desired oscillations and $x_n (t)$ for either $n=1$ or $n=2$. In step 2, “relaxation” (via $\delta (t)$) is used to address the hard constraints that $x_n (t) \ge 0$

Figure 2 displays parameterizeOscillator, our algorithm for designing an oscillator for the reaction network in Fig. 1. The python implementation of the algorithm is in the module designer.py in the github repository for this project. The implementation uses the python package lmfit to find $\hat{{\mathcal {P}}}_n (\theta ^{\star })$ using gradient descent.

There are some important details in the implementation of parameterizeOscillator. First, the quality of the optimization is greatly improved by doing multiple calls to lmfit using randomly chosen initial values for the independent parameters to more effectively scan the search space. Second, in calculating the loss functions, it is critical to adjust the density of time values (*t*) to the value of $\theta ^{\star }$ so that the algorithm sees at least two complete sinusoids at 10 or so different phases. Last, properly handling of the hard constraint $x_n(t) \ge 0$ is required so that gradient decent works effectively. We use relaxation [41], a technique that treats the hard constraints as soft constraints with large weights. This means that instead of using $L_n(({\mathcal {P}})$ in Eq. (12) as the loss function, we use $L^+_n({\mathcal {P}})$:

13 $$\begin{aligned} L_n^{+} ({\mathcal {P}}) = \sum _t \left( x^{\star }_n(t) -{\hat{x}}_n(t) \right) ^ 2 + \left( 1_{{\hat{x}}_1(t)} w {\hat{x}}_1 (t) \right) ^2 + \left( 1_{{\hat{x}}_2(t)} w {\hat{x}}_2 (t) \right) ^2 \end{aligned}$$

where $1_x$ is 1 if $x < 0$ and is 0 otherwise, and *w* is a large number. $L_n^{+} ({\mathcal {P}})$ has large gradients when $x_1(t) <0$ or $x_2(t) < 0$ so that gradient descent moves away from regions of the search space in which $C_2$ is violated.

Observe that parameterizeOscillator could be implemented using simulation instead of the formulas in Table 1. However, doing so would require considerably more computational resources. First, simulating the reaction network is several orders of magnitude slower than evaluating the formulas in Table 1. Second, the simulation approach does not exploit the relationships between parameters that reduce the size of the search space (e.g., calculating $k_d$ from $k_2, \theta ^{\star }$). Thus, a simulation-based algorithm would have a search space consisting of seven parameters in contrast to the 5 parameters used with the formulas based approach. This larger search space requires considerably more computational resources.

## Results

This Section analyzes the characteristics of the 2SHO and related analysis developed in the previous section.

### Accuracy of predictions of oscillation characteristics

**Fig. 3:**
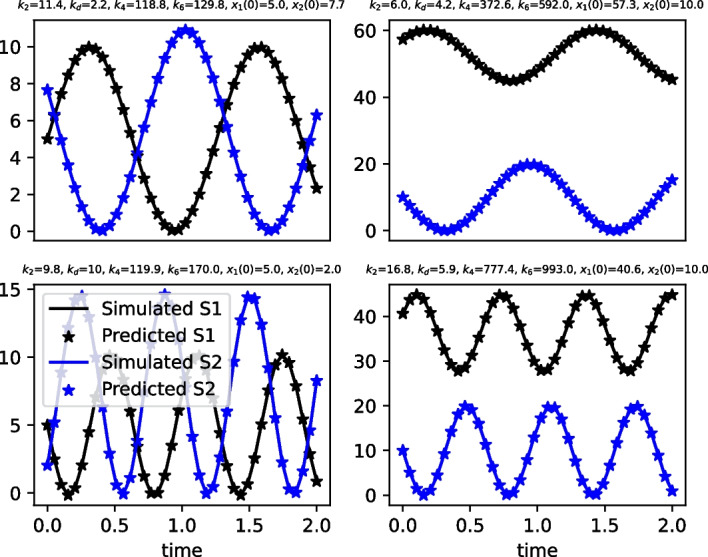
Evaluations of model accuracy. Plots of simulation results (solid lines) and model predictions (markers) using the formulas in Table 1 for four sets of parameter values of the reaction network in Fig. 1. In all cases, predictions coincide with the simulation results. The four cases have different values for the the independent parameters; dependent parameters are calculated as described in the text. The initial value of $S_n$ is $x_n (0)$

We begin by investigating the accuracy of predictions of 2SHO oscillation characteristics that are obtained from the formulas in Table 1. Figure 3 displays simulations of the 2SHO network detailed in Fig. 1 for four different values of the independent parameters. (Dependent parameters are calculated as described above.) Lines in the plots are simulation results, and the markers are model predictions using the formulas in Table 1. We see that in all cases, model predictions coincide with simulations results. We have done thousands of such simulations. In all cases, model predictions are identical with simulation results (within a tolerance of $10^{-13}$ to account for numerical errors). Although this does not prove the correctness of the model, it is strong confirmation. The ultimate proof is the correctness of our derivations as detailed in the Additional file 1.

Next, we investigate our algorithm for parameterizing the 2SHO as detailed in Fig. 2. Recall that the inputs to the algorithm are desired oscillation characteristics $\theta ^{\star }, \alpha ^{\star }, \phi ^{\star }, \omega ^{\star }$ that generate the sinusoidal concentrations $x^{\star }(t) = \alpha ^{\star } sin(\theta ^{\star } t + \phi ^{\star }) + \omega ^{\star }$. The algorithm finds independent parameters $\hat{{\mathcal {P}}} (\theta ^{\star })$ that generate the concentrations ${\hat{x}} (t)$ for one of $S_1, S_2$ with the constraint that there are no negative concentrations.

We use the term *design error* to refer to deviations between $x^{\star }(t)$ and ${\hat{x}}(t)$. We consider several kinds of design errors. A *feasibility design errors* occurs if ${\hat{x}}_n (t) < 0$ for some *n*, *t*. An *amplitude design error* is a deviation from $\alpha ^{\star }$. Let $\hat{\alpha }$ be the amplitude achieved by ${\hat{x}}(t).$ The amplitude design error is calculated as $\frac{\hat{\alpha } - \alpha ^{\star }}{\alpha ^{\star }}$. Similarly, *phase design error* is the deviation from $\phi ^{\star }$ of the phase (as a fraction of a cycle) achieved by $\hat{{\mathcal {P}}}(\theta )$. The phase design error is $\frac{\hat{\phi } - \phi ^{\star }}{2\pi }$. Note that we do not consider errors in the frequency that result from the parameters returned by the design algorithm. This is because the algorithm uses Eqs. (7), (3), and (4) to ensure that there are oscillating concentrations at the desired frequency $\theta ^{\star }$.

We simplify our studies by setting the desired DC offset $\omega ^{\star }$ to the desired oscillation amplitude $\alpha ^{\star }$. Our studies are conducted over 3 decimal orders of magnitude for both frequency (radians/sec) and amplitude: $\theta ^{\star }, \alpha ^{\star } \in [0.1, 100]$ in 8 increments. We consider four values of phase that are likely the most problematic because of challenges with calculating the inverse of the tangent function: $\phi ^{\star } \in \{0, \frac{\pi }{2}, \pi , \frac{2 \pi }{3} \}.$ Throughout, we set the maximum value of the kinetic constants ($k_i$) to 1000 to make the results more credible, but doing so can increase design errors.

We begin with feasibility design errors. parameterizeOscillator is very robust to feasibility design errors. We have conducted several thousand simulations, and only a couple of them returned values of independent parameters that had negative values for the concentrations of $S_1, S_2$.

**Fig. 4:**
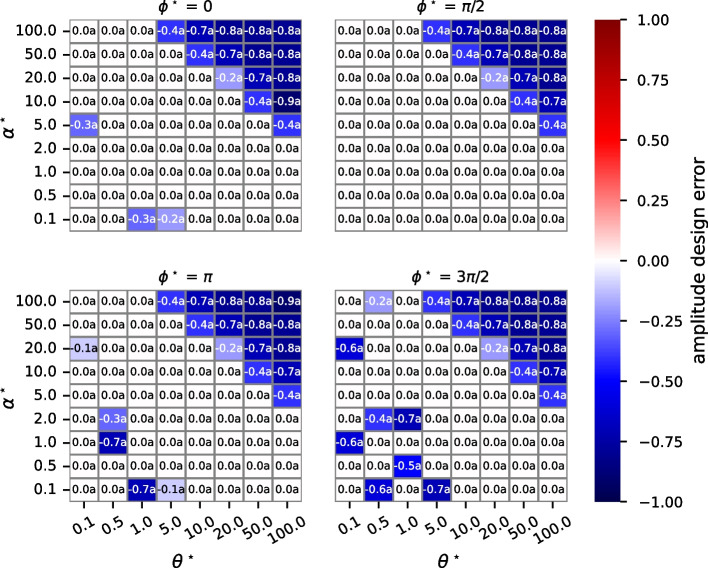
Amplitude design errors when parameterizeOscillator in Fig. 2 is modified so that $S_1$ is always the chosen oscillating species. The heatmaps display amplitude design errors for four phases ($\phi ^{\star }$). In each heatmap, the horizontal axis is desired frequency ($\theta ^{\star }$), and the vertical axis is desired amplitude ($\alpha ^{\star }$). Cell colors indicate the magnitude of the amplitude design error, and cells are annotated with the actual value. The letter “a” indicates that species $S_1$ is the chosen oscillating species

To analyze amplitude design errors, we first consider a variant of the algorithm in Fig. 2 in which the algorithm always returns $\hat{{\mathcal {P}}}_1 (\theta )$, the parameter estimates obtained if $S_1$ is the chosen oscillating species. Figure 4 displays the results of these studies. There are four plots, one for each value of phase. Each plot is a heatmap with desired frequency ($\theta ^{\star }$) as the horizontal axis and desired amplitude ($\alpha ^{\star }$) as the vertical axis. Cells are colored by the magnitude of the design error, and they are annotated with the value of the design error followed by a letter. The letter “a” means that $S_1$ is the chosen oscillating species; “b” indicates that $S_2$ is the chosen oscillating species. Only the letter “a” appears in these plots since $S_1$ is always the chosen oscillating species.

**Fig. 5:**
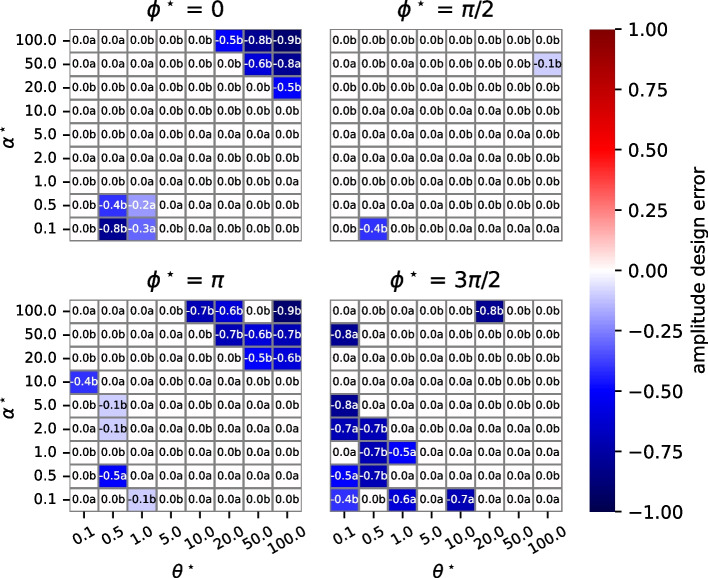
Amplitude design errors are reduced when parameterizeOscillator is used as-is. By so doing, either $S_1$ or $S_2$ can be the chosen oscillating species. The heatmaps are structured as in Fig. 4. The letter “a” indicates that species $S_1$ is the chosen oscillating species, and “b” indicates that $S_2$ is the chosen oscillating species

Amplitude design error is in the range $[-1, \infty ]$. A 0 means that there is no design error; a -1 means that the chosen oscillating species always has a concentration of zero (and so there is no oscillation). Amplitude design error is mostly 0 in the plots, except when both amplitude and frequency are large. The reason is that in our studies, the maximum value of kinetic constants is 1000. When both $\theta ^{\star }$ and $\alpha ^{\star }$ are large, much larger values of $k_4, k_6$ are required. Figure 5 plots the results of studies in which either $S_1$ or $S_2$ may be the chosen oscillating species, as is done in parameterizeOscillator. We see that there is a significant reduction in amplitude design error.

**Fig. 6:**
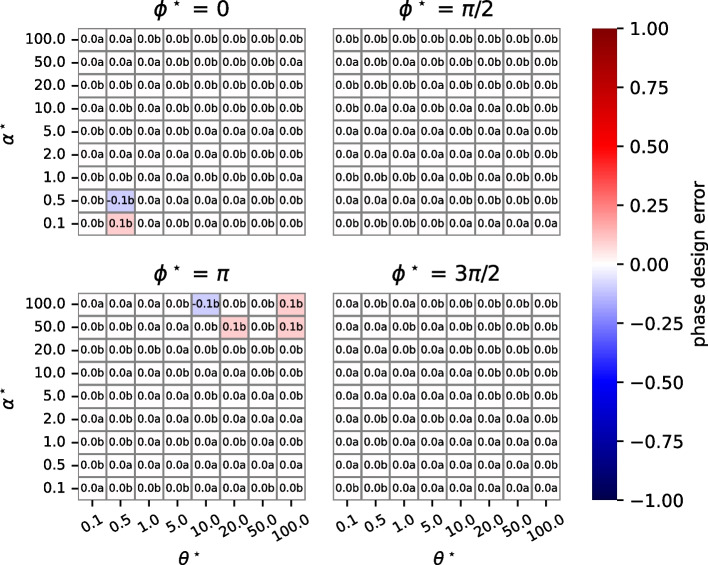
Phase design errors. These heatmaps are organized in the same way as Fig. 4, but cell values are phase design errors, the fraction of a cycle that the phase of the designed network differs from the phase of the desired oscillations. parameterizeOscillator almost always produces a phase design error of 0

Figure 6 displays the results for phase design error (in units of the fraction of an oscillation cycle). We see that phase design errors are mostly 0, although occasionally there is an error of 0.1 or $-$ 0.1.

**Fig. 7:**
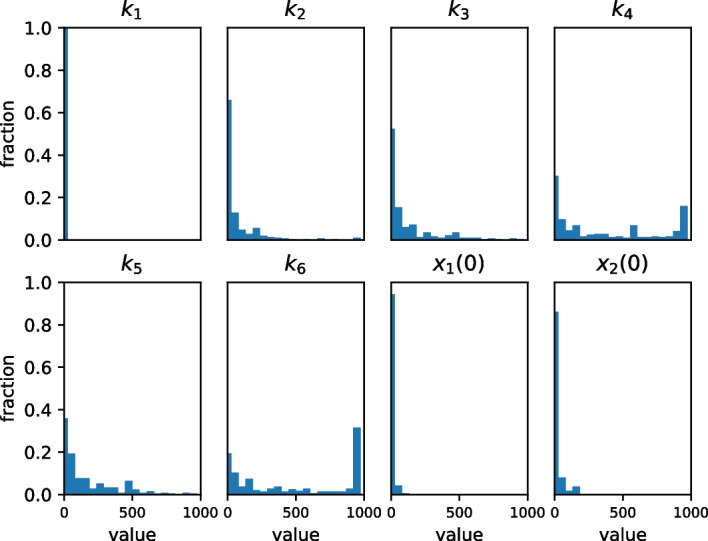
Histograms of values of parameter estimates in the numerical studies. The algorithm limits parameter values to the range [0, 1000]. Only the parameters associated with the boundary reactions $R_4$ ($k_4$) and $R_6$ ($k_6$) have values close to the upper bound of this range. These larger values are needed to construct oscillators that produce large amplitudes and high frequencies

Figure 7 studies the distribution of parameter values for the foregoing studies. Note that $k_1=1$ by design since from Table 1, we know that $k_1$ does not influence the behavior of the reaction network. Also, by design, the maximum value of a kinetic constant is 1000.

The parameters $k_2$, $k_3$, and $k_5$ are mass action kinetic constants for reactions with a single reactant. We see that they mostly have small values, although there are some instances in which these constants exceed 500. On the other hand, the zeroth order kinetic constants $k_4$ and $k_6$ tend to be much larger. Because our studies restrict parameterizeOscillator to choose $k_4, k_6$ with values less than 1000, there are larger amplitude design errors for studies in which both the frequency and amplitude are large.

### Parameter sensitivity and plausibility of biochemical implementation

Here we explore the sensitivity of the oscillation characteristics of 2SHO to variation in parameter values, especially with the thought of a biochemical implementation of the harmonic oscillator.

We start by analyzing the relative sensitivity of frequency, $\theta$, to variation in values of a parameter *k*. This relative sensitivity is denote by $S_k^{\theta }$. As defined in [42]:

$$\begin{aligned} S_k^{\theta } = \frac{\partial \theta }{\partial k} \frac{k}{\theta } \end{aligned}$$

Note that this is a unitless quantity. From Eq. (7), we know that $\theta = \sqrt{ k_2 k_d} = \sqrt{k_2 (k_5 - k_3)}$, and so:

14 $$\begin{aligned} S^{\theta }_{k_2 }= & {} \frac{1}{2} \end{aligned}$$

15 $$\begin{aligned} S^{\theta }_{k_3 }= & {} \frac{- k_{3}}{2 \left( k_{5} - k_{3} \right) } \end{aligned}$$

16 $$\begin{aligned} S^{\theta }_{k_5 }= & {} \frac{k_{5}}{2 \left( k_{5} - k_{3} \right) } \end{aligned}$$

$S^{\theta }_k = 0$ for $k \notin \{k_2, k_3, k_5 \}$. To put this in perspective, we likely want $k_5>> k_3$ to ensure that we get oscillations. So, for $k \in \{k_2, k_3, k_5 \}$, $|S^{\theta }_k| \approx \frac{1}{2}$. That is, there is a 0.5% change in $\theta$ for a 1% change in *k*.

The remaining OCs are complicated functions of the 2SHO parameters, and so are difficult to analyze analytically. Instead, we conduct numerical studies in which values of the 2SHO parameters are chosen randomly. The primary objective of these studies is to determine the feasibility of constructing a 2SHO for a biochemical system. The details of the biochemistry are beyond the scope of this paper. However, we do address various considerations for selection of the species and reactions since 2SHO imposes some constraints.

The first constraint comes from Eq. (3), that $k_3 + k_1 + k_2$. This constraint is difficult to achieved by a priori design because of the variability of values of kinetic constants. We propose addressing this constraint by having reaction $R_3$ be controlled enzymatically and operate in a region of low enzyme concentrations so that mass action kinetics are a good approximation. By titrating the enzyme, it should be possible to be fairly close to the Eq. (3) constraint. We refer to this as the *titration approach*.

A second constraint imposed by 2SHO is that $k_3 < k_5$. This constraint is easily realized by our choice of 2SHO parameters. Specifically, we use: $k_1=1.00,~ k_2=3.91,~ k_3=4.91, k_4=4.77,~ k_5=15.00,~ k_6=92.2,~ x_1(0)=5.00,~ x_2(0)=10.49$. This produces the OCs $\theta =6.28,~ \alpha _1=3.00,~ \alpha _2=5.67,~ \phi _1=0.00, \phi _2=2.12,~ \omega _1=5.00,~ \omega _2=5.67$. Clearly, $k_5 \gg k_3$.

The third constraint is that concentrations must be non-negative; that is, $\omega _n \ge \alpha _n$. Since this is a non-trivial constraint to assess, we analyze it numerically. We consider the values of the 2SHO parameters to be random variables whose distributions are centered on the parameters in the previous paragraph. Many have explored the experimental error associated with estimating kinetic constants (e.g., [35, 36]). From this literature, it seems that the standard deviations of these errors are in the range of 1–20% of the value of the kinetic constant. Thus, our studies use a *normalized standard deviation* (also known as the coefficient of variation), which is the measured standard deviation divided by the sample mean.

**Fig. 8:**
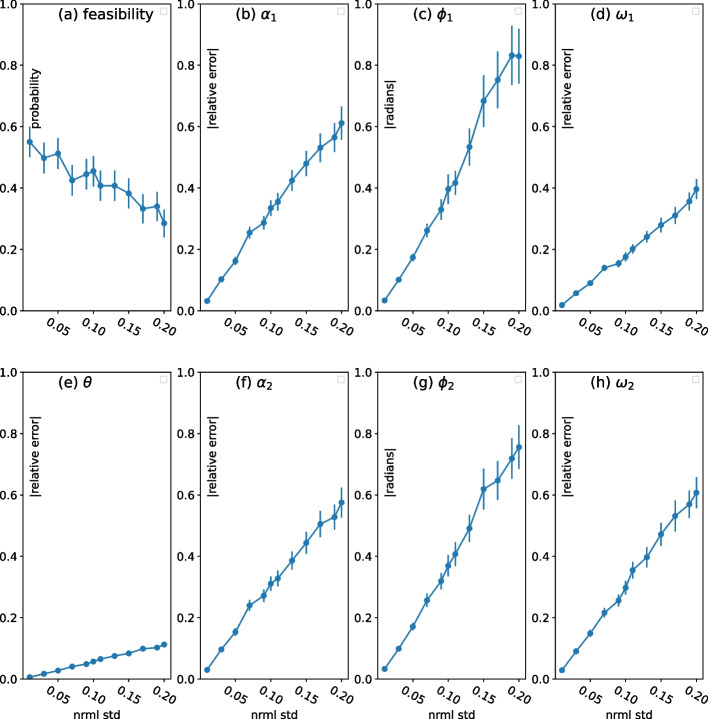
Sensitivity analysis of 2SHO. These plots show the effect of variability in the values of the parameters $k_1, k_2, k_4, k_5, k_6, x_1(0), x_2(0)$ on the ability to implement a 2SHO in a biochemical system. ($k_3 = k_1 + k_2$ by the design of the biochemical system.) Parameter values are drawn from a truncated normal distribution (no negative values) with n = 400 and whose mean is the desired value of the parameter and whose (normalized) standard deviation (coefficient of variation) is representative of empirical estimates of kinetic constants. Three kinds of metrics are displayed. Feasibility, chart (**a**), is the probability that a feasible oscillator results (e.g., no negative concentrations). Errors in expected values of OCs are relative to the desired value in charts (**b**, **d**, **e**, **f**, **h**). Discrepancies in phase, charts (**c**, **g**), are in units of radians. Error bars indicate ± two standard deviations

Figure 8a displays our results for feasibility, those situations when the randomly chosen parameters satisfy the constraints that $k_5 \ge k_3$ and $\omega _n > \alpha _n$. (Vertical bars $are \pm two$ standard deviations.) Feasibility is about 0.57 when normalized standard deviation is 0.01, and it declines to about 0.33 when normalized standard deviation is 0.2. Put differently, the probability *p*(*m*, *f*) of making a 2SHO in *m* tries with a feasibility of *f* for each try is $p(m,f) = 1-(1-f)^m$. Note that $p(4; 0.57) \approx 0.9 \approx p(6; 0.37)$. That is, based on the variability of estimates of kinetic constants, *there is a 90% chance of making a 2SHO in 4 to 6 attempts.*

Figure 8b–h display measures of the deviation of the oscillation characteristics of situations in which a feasible oscillator is obtained. For $\alpha _n, \omega _n$, we use the absolute value fractional deviation, $|{\hat{z}}-z|/z$, where ${\hat{z}}$ is the randomly chosen value that is centered at *z*. This doesn’t work for $\phi$, since $\phi _1=0$. So, for $\phi$, we do not divide by the desired value. As with feasibility, we see that the deviation from desired OCs increases with normalized standard deviation. Of the OCs, frequency $\theta$ seems to be affected the least.

## Discussion

This section explores the 2SHO in more detail using the formulas in Table 1.

We begin by examining the parameters of the reaction network. Consider $k_1$, the kinetic constant for reaction $R_1$. $k_1$ does not appear in Table 1. Indeed, the only reference to $k_1$ is in Eq. (3) to calculate $k_3$. So, $k_1$ can be any non-negative number. If $k_1= 0$, we have effectively eliminated reaction $R_1$. We have done many thousands of simulations in which $k_1 = 0$ with various values of the independent parameters and calculating the dependent parameters as described above. In all cases, we obtain the oscillating networks predicted by Table 1. From this we conclude, that $R_1$ is not required.

Next, consider $k_2$, the kinetic constant that controls the rate at which $S_2$ is converted to $S_1$. That is, because of reaction $R_2$, if $S_2$ is larger at time *t*, then $S_1$ will be larger at a future time, say $t + t_d$ for $t_d >0$. In essence, $k_2$ controls the phase shift from $S_2$ to $S_1$. We can see this using the results in Table 1. If $k_2$ is large, then the two species have the same phases: $lim_{k_2 \rightarrow \infty } \phi _1 = \frac{k_4 -k_6}{x_1(0) + x_2(0)} = lim_{k_2 \rightarrow \infty } \phi _2$. This phase shift in turn determines the frequency, since $\theta = \sqrt{k_2 k_d}.$ So, a large rate at which there is a change in phase in turn results in higher frequency oscillations.

Now consider $k_3, k_5$, the kinetic constants for reactions $R_3$ and $R_5$. $R_3$ is an autocatalysis reaction in which $S_1$ produces two copies of itself. This is a kind of positive feedback at the rate $k_3 S_1$. $R_5$ is degradation reaction in which $S_2$ is eliminated at the rate $k_5 S_1$. This is a kind of negative feedback in that a larger concentration of $S_1$ reduces the concentration of $S_2$, which in turn reduces the concentration of $S_1$ (because of $R_2$). From Eq. (4), we know that $k_5 > k_3$. Indeed, when $k_5 = k_3$, then the only eigenvalue of the system is 0. So, negative feedback must be larger than positive feedback in our 2SHO.

Reactions $R_4, R_6$ have zeroth order kinetics. These are essentially external tuning knobs that adjust oscillation characteristics in complex ways. An extreme case is in the DC offsets $\omega _n$: if $k_4 = 0 = k_6$, then $\omega _1 = 0 = \omega _2$. Note that $k_4, k_6$ appear in every formula for the OCs in Table 1 except frequency $\theta$.

Finally, we address the initial concentrations of $S_1, S_2$, which are denoted by $x_n(0)$. We see that initial concentrations affect amplitude, as is expected for a harmonic oscillator. We see that initial conditions also affect phase. However, initial concentrations do not impact frequency or DC offset.

Next we comment on related work in light of our methods and results. We start with claims related to nonlinearity. Gonze and Ruoff [30] claims that “the kinetic rate laws of the reaction mechanism must be sufficiently ‘nonlinear’ to destabilize the steady state.” This claim is echoed by [31] as well. Our 2SHO is a counter example to these claims in that its kinetics are described by a system of *linear* differential equations.

We construct 2SHO from two species because this simplifies the analysis for ensuring that the largest eigenvalue is a pure imaginary number. It is an open question if such a construction can be done for three or more species, and if so, can we obtain closed form solutions for the oscillation characteristics.

Another remark worthy of comment is that oscillations can result from a combination of positive and negative feedback [31]. Indeed, Fig. 1 has both positive and negative feedback. Reaction $R_3$ provides positive feedback at a rate $k_3 S_1$ through autocatalysis, and reaction $R_5$ provides negative feedback at the rate $k_5 S_1$ by degrading $S_2$. We add to these remarks the observation that the rate of negative feedback must be larger than the rate of positive feedback, at least in our reaction network, since by Eq. (4), $k_5 > k_3.$

Our final remark is a bit more speculative. Disciplines such as electrical and mechanical engineering make extensive use of system identification and control theory in their designs and analyses. A key element of these techniques is the use of frequency analysis such as Bode plots. Frequency analysis requires the ability to generate sinusoids with specific oscillation characteristics, especially frequency, amplitude, and phase. While others have demonstrated the construction of biological clocks (e.g., [43, 44]), there is no technique for generating sinusoids with arbitrary oscillation characteristics. Our parameterizeOscillator algorithm provides a way to choose values of the parameters of the reaction network in Fig. 1 to achieve desired The implementation of a biological signal generator is beyond the scope of this paper. However, such an implementation likely requires enzyme engineering techniques such as those discussed in [40].

**Table 1:**
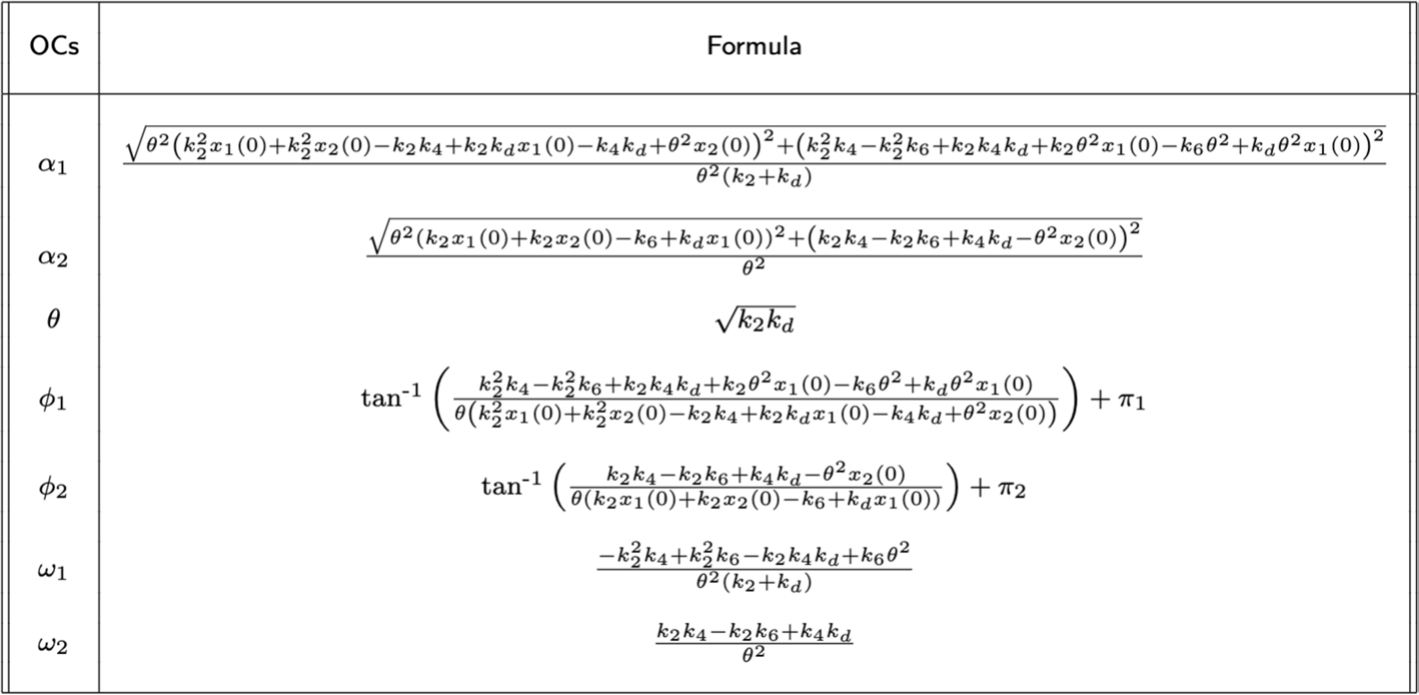
Formulas for oscillator characteristics (OCs)

OCs are expressed in terms of the kinetic constants $k_i$ of the reaction network in Fig. 1 and the initial concentrations of the chemical species, $x_n(0)$ for species $S_n$. The formulas are obtained by solving the system of equations for the reaction network. The oscillator characteristics (OCs) are: amplitude ($\alpha _n$), frequency ($\theta$), phase ($\phi _n$), and DC offset ($\omega _n$). The terms $\pi _1, \pi _2$ are defined in the text, and reflect technical details related to the inverting the tangent function

## Conclusions

Oscillatory behavior is critical to many life sustaining processes, such as cell cycles, circadian rhythms, and notch signaling. We use the term oscillation characteristics (OCs) to refer to the frequency, amplitude, phase, and DC offset of oscillations. OCs affect many biological functions. Examples include: the timing of event initiations can be controlled by oscillation frequency; the strength of signaling can be regulated by the amplitude of oscillations; and the sequence of events can be determined by the phase of oscillations.

This paper is a theoretical study that shows that sustained oscillations are possible in a reaction network with linear dynamics. By linear dynamics is meant that the rate laws are linear in species concentrations. It has not been obvious to many that such networks can produce oscillations. Gonze and Ruoff [30] claim that oscillations in reaction networks require that “the kinetic rate laws of the reaction mechanism must be sufficiently ‘nonlinear’ to destabilize the steady state. ” This claim is echoed by [31] as well. We note that the context for these claims may be for biological implementations of oscillators. Even so, the question remains, can oscillations be generated by a reaction network with linear dynamics?

Although it is fairly staright-forward to create a system of linear ODEs that oscillates, it is non-trivial to create an oscillating *reaction network* with linear dynamics. The challenges are: (a) species concentrations must be non-negative; (b) the semantics of reactions imposes constraints on the Jacobian matrix (e.g., a single reaction can affect multiple entries in the matrix); and (c) reaction rate laws must be biologically plausible.

Our first contribution is the construction of a reaction network that implements a two species harmonic oscillator (2SHO). This is shown in Fig. 1. We are unaware of others who have implemented a harmonic oscillator in a reaction network. The dynamics of 2SHO are described by a system of linear differential equations. We note that [30, 31] claim that oscillating reaction networks require nonlinearities. This may be true for biological implementations, but it certainly is not true in theory for a reaction network.

Our second contribution is obtaining closed form formulas that calculate the oscillation characteristics (OCs) frequency, amplitude, and phase of the 2SHO in terms of its parameters. The formulas are displayed in Table 1. We know of no other oscillating reaction network for which such formulas have been obtained.

Our third contribution is the development of an algorithm that finds values of parameters that result in a reaction network with desired OCs. The algorithm is detailed in Fig. 2. The algorithm makes use of the OC formulas. We show that the algorithm performs well over a wide range of OCs.

We further analyze the sensitivity of 2SHO to variations in values of the kinetic constants; typical varaiabilities are between 1 and 20%. Our analysis suggests that with 4 to 6 attempts, there is a 90% probability of constructing a 2SHO for a suitable biochemical system.

The OC formulas also allow us to comment on and/or extend other studies of oscillatory reaction networks. One theme in the literature is that nonlinear dynamics are required to create oscillations in reaction networks. Our 2SHO is a counter example to these claims in that its kinetics are described by a system of linear differential equations. Another observation in the literature is that an oscillating network can be constructed by combining positive and negative feedback, an approach used in 2SHO as well. Our formulas extend this insight by showing that for 2SHO, the rate of negative feedback must exceed the rate of positive.

We are very interested in exploring how our results for designing an oscillating linear network might be applied to designing an oscillating nonlinear network. One direction here is using 2SHO to construct a linear approximation to the nonlinear network. If we can develop an appropriate mapping between the two networks, we can tune the linear network in a desired way, and then map these adjustments back to the nonlinear network.

### Supplementary Information

**Additional file 1**. Supplementary material: Detailed derivations.

## Data Availability

Supplementary material is available at https://github.com/ModelEngineering/Oscillators/blob/main/docs/supplemental_material/supplemental_material.pdf, and the github project for this paper is at https://github.com/ModelEngineering/Oscillators.

## Abbreviations

2SHO: Two species harmonic oscillator
OC: Oscillation characteristic
ODE: Ordinary differential equation
$${\textbf {A}}$$: Jacobian matrix
$\alpha _n$: Amplitude of oscillation for species *n*
$$\alpha _n^{\star }$$: Desired amplitude
$\hat{\alpha }_n$: Amplitude of the oscillation produced by parameterizeOscillator
$${\textbf {c}} = \begin{pmatrix} c_1 \\ c_2 \end{pmatrix}$$: Constants associated with the homogeneous solution
$$\Delta$$: $$det {\textbf {A}})$$ (determinant)
$${\textbf {F}} (t)$$: Fundamental matrix
*i*: Indexes kinetic constants
$k_i$, $k_d$: Non-negative constants for kinetic constants
$$\lambda _n$$: Eigenvalue
*n*: Indexes states (chemical species)
*N*: Number of species
$\omega _n$: DC offset of species *n*
$\omega _n^{\star }$: Desired DC offset
$\hat{\omega }_n$: DC offset of the oscillation produced by parameterizeOscillator
${\mathcal {P}}$: The set of parameters and associated values for the reaction network in Fig. 1
${\mathcal {P}} (\theta )$: The set of parameters with $k_d$ determined by $\theta$ and $k_2$
$\phi _n$: Phase in radians for species *n*
$$\phi _n^{\star }$$: Desired phase
$\hat{\phi }_n$: Phase of the oscillation produced by parameterizeOscillator
$S_n$: Chemical species in the reaction network
*t*: Time
$$\tau$$: $$tr({\textbf {A}})$$ (trace)
$\theta$: Frequency in radians
$$\theta ^{\star }$$: Desired frequency
$\hat{\theta }$: Frequency of the oscillation produced by parameterizeOscillator
$${\textbf {u}} = \begin{pmatrix} u_1 \\ u_2 \end{pmatrix}$$: Forced input (kinetic constants for zeroth order rates)
$\dot{\textbf {x}} (t)$: Derivative w.r.t. time of ${\textbf {x}}$
$x_n(t)$: Time varying concentration of species *n*
$$x_n^{\star }$$: Desired signal
${\hat{x}}_n$: Signal produced the oscillator constructed parameterizeOscillator
