# Supplementary material for: An oscillating reaction network with an exact closed form solution in the time domain
Source: BMC Bioinformatics. 2023 Dec 9;24:466. doi: 10.1186/s12859-023-05600-w (PMC10710734; doi:10.1186/s12859-023-05600-w)
Supplement: Supplementary file 1 — Additional file 1. Supplementary material: Detailed derivations. [file 12859_2023_5600_MOESM1_ESM.pdf]

An Oscillating Reaction Network With an Exact Closed Form Solution in the Time Domain: Solution Derivation

Joseph L. Hellerstein
eScience Institute
University of Washington
Seatttle, Washington

1 Preliminaries

This write-up provides details on the derivation of the time domain solution for the two species harmonic oscillator reaction network depicted in Fig. 1. Recall that the reaction network is constructed so that its kinetics are described by a system of linear ODEs with sustained oscillations. The time domain solution is an initial value problem (IVP).

The reaction network has 8 parameters. There are 6 kinetic constants, $k_i,\ i \in \{1, 2, 3, 4, 5, 6\}$. And there are two initial concentrations, $x_n(0),\ n \in \{1, 2\}$, where $x_n(0)$ is the initial concentration for S_n.

Because the dynamics of the network can be described by an oscillating linear system, we know that oscillations are sinusoids. Let $x_n(t)$ be the concentration of species S_n at time t. Then, an oscillating solution has the form

$$x_n(t) = \alpha_n sin(\theta_n t + \phi_n) + \omega_n,$$

where α_n is the amplitude of the sinusoid for S_n, θ_n is its frequency, ϕ_n is its phase, and ω_n is the DC offset (the mean value of the sinusoid over time). As we show shortly, both species have the same frequency. So, $\theta_n = \theta$, and there are just 7 parameters. We refer to $\alpha_n, \theta, \phi_n, \omega_n$ as the **oscillation characteristics (OC)** of the reaction network.

We make use of the vector representation of linear systems of ODEs.

$$\dot{\mathbf{x}} = \mathbf{A}\mathbf{x} + \mathbf{u}$$

where the Jacobian matrix is $\mathbf{A} = \begin{pmatrix} a_{11} & a_{12} \\ a_{21} & a_{22} \end{pmatrix}$, $\mathbf{x}(t)$ is the state vector, $\dot{\mathbf{x}}(t)$ is the time derivative of the state vector, and $\mathbf{u}$ is the time-independent forced inputs.

2 Jacobian and Eigenvalues

Here, we construct the Jacobian of the reaction network, and calculate its eigenvalues.

If there is an oscillating solution for this system, then the eigenvalues of $\mathbf{A}$ must be pure imaginary. Since this is a two state system, this means that if θi is an eigenvalue, then $-\theta i$ must also be an eigenvalue. This means that $\theta_1 = \theta = \theta_2$, which justifies our previous claim that the two species have the same oscillation frequency.

Next we develop the conditions for the Jacobian $\mathbf{A}$ to have a pure imaginary eigenvalues. The determinant of $\mathbf{A}$ is $det(\mathbf{A}) = a_{11}a_{22} - a_{12}a_{21} = \Delta$. The trace of $\mathbf{A}$ is $tr(\mathbf{A}) = a_{11} + a_{22} = \tau$. So, the eigenvalues λ_n are $\frac{1}{2}\left(-\tau \pm \sqrt{\tau^2 - 4\Delta}\right)$. We get pure imaginary eigenvalues if the following constraints hold:

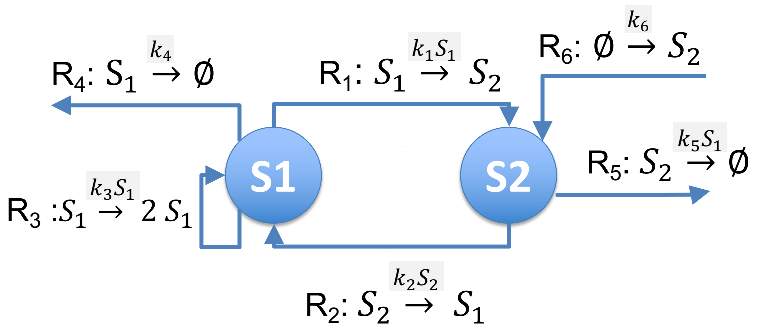

Figure 1 Reaction network that creates oscillations in the chemical species S_1, S_2. The reaction network is designed so that its time domain solution is a system of linear differential equations. The text describes constraints on the kinetic constants (k_i) and initial conditions of the chemical species to create an oscillator such that species concentrations are non-negative, a requirement for biological feasibility.

- $\tau = 0$
- $\Delta > 0$.

For the reaction network, $\mathbf{A}$ is

$$\mathbf{A} = \begin{pmatrix} k_3 - k_1 & k_2 \\ k_1 - k_5 & -k_2 \end{pmatrix}$$

and

$$\mathbf{u} = \begin{pmatrix} -k_4 \\ k_6 \end{pmatrix}$$

So the trace and determinant are:

$$\begin{aligned} \tau &= k_3 - k_1 - k_2 \\ \Delta &= (k_3 - k_1)(-k_2) - k_2(k_1 - k_5) \\ &= k_2(k_5 - k_3) \end{aligned}$$

$\tau = 0$ implies that $k_3 = k_1 + k_2$. $\Delta > 0$ implies that that $k_5 > k_3$. We define $k_d = k_5 - k_3 > 0$ Applying the foregoing to the **A** matrix, we first note that

$$\begin{aligned} k_1 - k_5 &= k_1 - k_3 - k_d \\ &= k_3 - k_2 - k_3 - k_d \\ &= -k_2 - k_d \end{aligned}$$

Applying the constraints, we see that

$$\mathbf{A} = \begin{pmatrix} k_2 & k_2 \\ -k_2 - k_d & -k_2 \end{pmatrix}$$

Observe that $\Delta = k_2 k_d$. As a result $\theta = \pm\sqrt{\Delta} = \pm\sqrt{k_2 k_d}$. The negative sign affects phase, not frequency; we address phase separately. So, hereafter, we drop the $\pm$.

3 Eigenvectors and Fundamental Matrix

We find the eigenvectors of **A** as an intermediate step to solving the IVP.

First, observe that that since $k_d > 0$, **A** is nonsingular, and so we can calculate eigenvectors directly.

$$\mathbf{w}_1 = \begin{pmatrix} -\frac{k_2}{k_2+k_d} + \frac{i\theta}{k_2+k_d} \\ 1 \end{pmatrix}$$

for the eigenvalue $\lambda_1 = -\sqrt{k_d k_2}$.

$$\mathbf{w}_2 = \begin{pmatrix} -\frac{k_2}{k_2+k_d} - \frac{i\theta}{k_2+k_d} \\ 1 \end{pmatrix}$$

for the eigenvalue $\lambda_2 = \sqrt{k_d k_2}$.

The **fundamental matrix F** of a system of linear ODEs has a column for each $\mathbf{w}_n e^{\lambda_n}$. Rather than writing $\mathbf{F}(t)$ immediately, we first observe that it can be transformed into a real valued matrix. This involves factoring the eigenvectors into real and imaginary parts and then applying Euler formulas to express $\mathbf{F}(t)$ in terms of *sin* and *cos*. For $\mathbf{w}_1[0]$ (the first row of this term), this means

$$\begin{aligned} \mathbf{w}_1 e^{\lambda_1}[0] &= \left(-\frac{k_2}{k_2+k_d} + \frac{i\theta}{k_2+k_d}\right)(cos(t\theta) - isin(t\theta)) \\ &= -\frac{k_2}{k_2+k_d}cos(t\theta) + \frac{\theta}{k_2+k_d}sin(t\theta) + i\left(\frac{k_2}{k_2+k_d}sin(t\theta) + \frac{\theta}{k_2+k_d}cos(t\theta)\right) \end{aligned}$$

and so

$$\mathbf{w}_1 e^{\lambda_1} = \begin{pmatrix} -\frac{k_2}{k_2+k_d}cos(t\theta) + \frac{\theta}{k_2+k_d}sin(t\theta) + i\left(\frac{k_2}{k_2+k_d}sin(t\theta) + \frac{\theta}{k_2+k_d}cos(t\theta)\right) \\ cos(t\theta) - isin(t\theta) \end{pmatrix}$$

Similarly,

$$\mathbf{w}_2 e^{\lambda_2} = \begin{pmatrix} -\frac{k_2}{k_2+k_d} cos(t\theta) + \frac{\theta}{k_2+k_d} sin(t\theta) - i\left(\frac{k_2}{k_2+k_d} sin(t\theta) + \frac{\theta}{k_2+k_d} cos(t\theta)\right) \\ cos(t\theta) + isin(t\theta) \end{pmatrix}$$

for the matrix

$$\mathbf{F}(t) = \begin{pmatrix} \mathbf{w}_1 e^{\lambda_1} & \mathbf{w}_2 e^{\lambda_2} \end{pmatrix}.$$

We do some further simplifications by taking linear combinations of the columns of the fundamental matrix, transformations that change the basis for the solution space but not the solution space itself. The fundamental matrix is constructed by having the first column as $\left(\mathbf{w}_1 e^{\lambda_1} + \mathbf{w}_2 e^{\lambda_2}\right)/2$ and the second column as $i\left(\mathbf{w}_1 e^{\lambda_1} - \mathbf{w}_2 e^{\lambda_2}\right)/2$.

$$\mathbf{F}(t) = \begin{pmatrix} -\frac{k_2 \cos(t\theta)}{k_2+k_d} + \frac{\theta \sin(t\theta)}{k_2+k_d} & -\frac{k_2 \sin(t\theta)}{k_2+k_d} - \frac{\theta \cos(t\theta)}{k_2+k_d} \\ \cos(t\theta) & \sin(t\theta) \end{pmatrix}$$

4 Solving the Initial Value Problem

We proceed in the usual way to solve an IVP:

1. Find the solution to the homogeneous system $\dot{\mathbf{x}}^H(t) = \mathbf{A}\mathbf{x}^H(t)$.
2. Find a particular solution such that $\dot{x}^P(t) = \mathbf{A}\mathbf{x}^P(t) + \mathbf{u}$.
3. Construct the full solution $\mathbf{x}(t) = \mathbf{x}^H(t) + \mathbf{x}^P(t)$.

$\mathbf{x}^H(t) = \mathbf{F}(t)\mathbf{c}$, where $\mathbf{c}$ is a vector of unknown constants that are determined based on initial conditions (where the initial conditions are the initial concentrations of S_1, S_2).

We find $\mathbf{x}^P(t)$ using the fundamental matrix. We assume that $\mathbf{x}^P(t) = \mathbf{F}(t)\mathbf{v}$ for some unknown $\mathbf{v}$, and we solve for this vector. Substituting for $\mathbf{x}^P(t)$ and recalling that each column of $\mathbf{F}(t)$ is a solution to the homogeneous system, we have

$$\begin{aligned} \dot{\mathbf{x}}^P(t) &= \mathbf{A}\mathbf{x}^P(t) + \mathbf{u} \\ \dot{\mathbf{F}}(t)\mathbf{v} + \mathbf{F}(t)\dot{\mathbf{v}} &= \mathbf{A}\mathbf{F}(t)\mathbf{v} + \mathbf{u} \\ \mathbf{A}\mathbf{F}(t)\mathbf{v} + \mathbf{F}(t)\dot{\mathbf{v}} &= \mathbf{A}\mathbf{F}(t)\mathbf{v} + \mathbf{u} \\ \mathbf{F}(t)\dot{\mathbf{v}} &= \mathbf{u} \end{aligned}$$

$$\mathbf{v} = \int \left(\mathbf{F}^{-1}(t)\mathbf{u}\right) dt$$

Calculating this integral (with help from the python package `sympy`), we have

$$\begin{aligned} \mathbf{x}^P(t) &= \mathbf{F}(t)\mathbf{v} \\ &= \begin{pmatrix} \frac{-k_2^2 k_4 \cos(t\theta) - k_2^2 k_4 + k_2^2 k_6 \cos(t\theta) + k_2^2 k_6 - k_2 k_4 k_d \cos(t\theta) - k_2 k_4 k_d + k_2 k_4 \theta \sin(t\theta) - k_2 k_6 \theta \sin(t\theta) + k_4 k_d \theta \sin(t\theta) + k_6 \theta^2}{\theta^2 (k_2 + k_d)} \\ \frac{k_2 k_4 \cos(t\theta) + k_2 k_4 - k_2 k_6 \cos(t\theta) - k_2 k_6 + k_4 k_d \cos(t\theta) + k_4 k_d}{\theta^2} \end{pmatrix} \end{aligned}$$

$$
\begin{aligned}
\mathbf{x}(t) &= \mathbf{x}^H(t) + \mathbf{x}^P(t) \\
&= \begin{pmatrix} -\frac{k_2\cos(t\theta)}{k_2+k_d} + \frac{\theta\sin(t\theta)}{k_2+k_d} & -\frac{k_2\sin(t\theta)}{k_2+k_d} - \frac{\theta\cos(t\theta)}{k_2+k_d} \\ \cos(t\theta) & \sin(t\theta) \end{pmatrix} \begin{pmatrix} c_1 \\ c_2 \end{pmatrix} \\
&\quad + \begin{pmatrix} \frac{-k_2^2k_4\cos(t\theta) - k_2^2k_4 + k_2^2k_6\cos(t\theta) + k_2^2k_6 - k_2k_4k_d\cos(t\theta) - k_2k_4k_d + k_2k_4\theta\sin(t\theta) - k_2k_6\theta\sin(t\theta) + k_4k_d\theta\sin(t\theta) + k_6\theta^2}{\theta^2(k_2+k_d)} \\ \frac{k_2k_4\cos(t\theta) + k_2k_4 - k_2k_6\cos(t\theta) - k_2k_6 + k_4k_d\cos(t\theta) + k_4k_d}{\theta^2} \end{pmatrix}
\end{aligned}
$$

We find the constants c_1, c_2 by solving the linear system below. (Recall that $x_n(0)$ is the initial concentration of S_n, which is an input to the IVP.)

$$
\begin{pmatrix} x_1(0) \\ x_2(0) \end{pmatrix} = \begin{pmatrix} x_1(t)_{t=0} \\ x_2(t)_{t=0} \end{pmatrix} \begin{pmatrix} c_1 \\ c_2 \end{pmatrix}
$$

The resulting equations are lengthy, and so we display the $x_n(t)$ separately.

$$
\begin{aligned}
x_1(t) &= \frac{1}{\theta}\left(-\frac{k_2\sin(t\theta)}{k_2+k_d} - \frac{\theta\cos(t\theta)}{k_2+k_d}\right)(-k_2x_1(0) - k_2x_2(0) + k_6 - k_dx_1(0)) \qquad (1) \\
&+ \frac{1}{\theta^2}\left(-\frac{k_2\cos(t\theta)}{k_2+k_d} + \frac{\theta\sin(t\theta)}{k_2+k_d}\right)\left(-2k_2k_4 + 2k_2k_6 - 2k_4k_d + \theta^2x_2(0)\right) \\
&+ \frac{1}{\theta^2(k_2+k_d)}\left(-k_2^2k_4\cos(t\theta) - k_2^2k_4 + k_2^2k_6\cos(t\theta) + k_2^2k_6 + k_6\theta^2 - k_2k_4k_d\right) \\
&+ \frac{1}{\theta^2(k_2+k_d)}\left(-k_2k_4k_d\cos(t\theta) + k_2k_4\theta\sin(t\theta) - k_2k_6\theta\sin(t\theta) + k_4k_d\theta\sin(t\theta)\right)
\end{aligned}
$$

$$
\begin{aligned}
x_2(t) &= \frac{1}{\theta}(-k_2x_1(0) - k_2x_2(0) + k_6 - k_dx_1(0))\sin(t\theta) \qquad (2) \\
&+ \frac{1}{\theta^2}\left(-2k_2k_4 + 2k_2k_6 - 2k_4k_d + \theta^2x_2(0)\right)\cos(t\theta) \\
&+ \frac{1}{\theta^2}(k_2k_4\cos(t\theta) + k_2k_4 - k_2k_6\cos(t\theta) - k_2k_6 + k_4k_d\cos(t\theta) + k_4k_d)
\end{aligned}
$$

5 Formulas for Oscillation Characteristics

Our final task is to restructure $\mathbf{x}(t)$ to isolate the oscillation characteristics $\theta, \alpha_n, \phi_n, \omega_n$. The first step is to factor Eq. (1) and Eq. (2) so that

$$
x_n(t) = a_n\cos(\theta t) + b_n\sin(\theta t) + \omega_n \qquad (3)
$$

Observe that ω_n (the DC offset) is the sum of terms that are *not* multiplied by $\cos(t\theta)$ or $\sin(t\theta)$. From Eq. (3), we calculate α_n, ϕ_n using the the trigonometric equality $a_n cos(t\theta) + b_n sin(t\theta) = \sqrt{a_n^2 + b_n^2} sin(t\theta + tan^{-1}\frac{a_n}{b_n})$. Thus, $\alpha_n = \sqrt{a_n^2 + b_n^2}$, and $\phi_n = tan^{-1}\frac{a_n}{b_n}$.

The results are:

- $\theta = \sqrt{k_2k_d}$

- $\alpha_1 = \frac{\sqrt{\nu_1}}{\theta^2(k_2+k_d)}$

$$\begin{aligned}\nu_1 &= \theta^2\left(k_2^2x_1(0)+k_2^2x_2(0)-k_2k_4+k_2k_dx_1(0)-k_4k_d+\theta^2x_2(0)\right)^2\\ &+\left(k_2^2k_4-k_2^2k_6+k_2k_4k_d+k_2\theta^2x_1(0)-k_6\theta^2+k_d\theta^2x_1(0)\right)^2\end{aligned}$$

- $\alpha_2 = \frac{\sqrt{\theta^2(k_2x_1(0)+k_2x_2(0)-k_6+k_dx_1(0))^2+(k_2k_4-k_2k_6+k_4k_d-\theta^2x_2(0))^2}}{\theta^2}$
- $\phi_1 = \tan^{-1}\left(\frac{k_2^2k_4-k_2^2k_6+k_2k_4k_d+k_2\theta^2x_1(0)-k_6\theta^2+k_d\theta^2x_1(0)}{\theta\left(k_2^2x_1(0)+k_2^2x_2(0)-k_2k_4+k_2k_dx_1(0)-k_4k_d+\theta^2x_2(0)\right)}\right)+\pi_1$
- $\phi_2 = \tan^{-1}\left(\frac{k_2k_4-k_2k_6+k_4k_d-\theta^2x_2(0)}{\theta(k_2x_1(0)+k_2x_2(0)-k_6+k_dx_1(0))}\right)+\pi_2$
- $\omega_1 = \frac{-k_2^2k_4+k_2^2k_6-k_2k_4k_d+k_6\theta^2}{k_2\theta^2+k_d\theta^2}$
- $\omega_2 = \frac{k_2k_4-k_2k_6+k_4k_d}{\theta^2}$

π_1, π_2 arise from techical conditions related to taking the inverse of the tangent function. Tangent is only defined over a range of π since it is calculated from the ratio of a_n to b_n. Hence, we cannot distinguish between two positive values of these number, and two negative values of these numbers.

$$\begin{aligned}cond_1 &= \frac{k_2^2x_1(0)}{k_2\theta+k_d\theta}+\frac{k_2^2x_2(0)}{k_2\theta+k_d\theta}+\frac{k_2k_4\theta}{k_2\theta^2+k_d\theta^2}-\frac{2k_2k_4}{k_2\theta+k_d\theta}-\frac{k_2k_6\theta}{k_2\theta^2+k_d\theta^2}\\ &+\frac{k_2k_6}{k_2\theta+k_d\theta}+\frac{k_2k_dx_1(0)}{k_2\theta+k_d\theta}+\frac{k_4k_d\theta}{k_2\theta^2+k_d\theta^2}-\frac{2k_4k_d}{k_2\theta+k_d\theta}+\frac{\theta x_2(0)}{k_2+k_d}\end{aligned}$$

$$\begin{aligned}\pi_1 &= \pi \text{ if } cond_1 < 0\\ &= 0 \text{ otherwise}\end{aligned}$$

$$cond_2 = \frac{k_2x_1(0)}{\theta}+\frac{k_2x_2(0)}{\theta}-\frac{k_6}{\theta}+\frac{k_dx_1(0)}{\theta}$$

$$\begin{aligned}\pi_2 &= \pi \text{ if } cond_2 > 0\\ &= 0 \text{ otherwise}\end{aligned}$$

Notation

- $\mathbf{A}$ - Jacobian matrix
- α_n - amplitude of oscillation for species n
- $\mathbf{c} = \begin{pmatrix} c_1 \\ c_2 \end{pmatrix}$ - constants associated with the homogeneous solution
- Δ - $det\mathbf{A}$) (determinant)
- $\mathbf{F}(t)$ - fundamental matrix
- i - indexes kinetic constants
- k_i, k_d - non-negative constants for kinetic constants
- λ - eigenvalue
- n - indexes states (chemical species)
- N - number of species

- ω_n - DC offset of species n
- ϕ_n - phase in radians
- S_n - chemical species in the reaction network
- t - time
- τ - $tr(\mathbf{A})$ (trace)
- θ - frequency in radians
- $\mathbf{u} = \begin{pmatrix} u_1 \\ u_2 \end{pmatrix}$ - forced input (kinetic constants for zeroth order rates)
- $\dot{\mathbf{x}}(t)$ - derivative w.r.t. time of $\mathbf{x}$
- $x_n(t)$ - time varying concentration of species n
